# Lack of Association between Hsa-miR-149 rs2292832 Polymorphism and Cancer Risk: A Meta-Analysis of 12 Studies

**DOI:** 10.1371/journal.pone.0073762

**Published:** 2013-09-05

**Authors:** Lei Xu, Xin Zhou, Man-Tang Qiu, Rong Yin, Ya-Qin Wu, Lin Xu

**Affiliations:** 1 The Fourth Clinical College of Nanjing Medical University, Nanjing, China; 2 Department of Thoracic Surgery, Nanjing Medical University Affiliated Cancer Hospital Cancer Institute of Jiangsu Province, Nanjing, China; 3 Department of Oncology, First Clinical College of Nanjing Medical University, Nanjing, China; ENEA, Italy

## Abstract

**Background:**

MicroRNAs (miRNAs) participate in various cellular processes such as cell growth, differentiation, cell death and play an important role in a variety of diseases, especially in cancer. Recently, a number of studies have investigated the association between single nucleotide polymorphisms (SNPs) on the hsa-miR-149 rs2292832 and susceptibility to cancer; however, the results remain inconclusive.

**Methodology/Principal Findings:**

We carried out a meta-analysis of 12 studies including 5937 cases and 6081 controls from PubMed to assess the association between the hsa-miR-149 rs2292832 and cancer risk by pooled odds ratios (ORs) and 95% confidence intervals (CIs). However, our results showed that genotype distribution of the hsa-miR-149 rs2292832 was not associated with cancer risk in all genetic models. Subgroup analysis by cancer type, ethnicity or study design showed no significant association either.

**Conclusion:**

Results of this meta-analysis suggest that the hsa-miR-149 rs2292832 polymorphism is not associated with cancer risk in spite of the potentially protective role of C allele in hepatocellular carcinoma and male gastric cancer.

## Introduction

MicroRNAs (miRNAs) are an abundant class of small non-coding RNAs that negatively regulate gene expression by base pairing with the 3’-untranslated region of target mRNAs, resulting in either mRNA cleavage or translational repression [1,2]. Many studies have indicated that miRNAs are involved in regulating various biological processes, such as cellular differentiation, proliferation, angiogenesis, metabolism and cancer development [3–7]. As a crucial part of tumor formation, maintenance, and metastasis, dysregulated miRNAs, as either tumor suppressors or oncogenes, may play an important role in cancer [8].

Single nucleotide polymorphisms (SNPs) located at miRNA genes may be associated with changes in miRNA processing, thus leading to functional changes of miRNA by influencing interaction between miRNAs and their target mRNAs [9,10]. Many studies have explored association between SNPs of miRNA and susceptibility to various cancers. The hsa-miR-499 rs3746444 polymorphism might increase breast cancer risk [11]. Wang et al. showed that the miR-146a rs2910164 polymorphism was significantly associated with risk of papillary thyroid carcinoma, primary liver cancer and cervical cancer and the miR-196a2 rs11614913 polymorphism was associated with breast cancer, lung cancer, and colorectal cancer [12].

Recently, many studies have investigated the association between the hsa-miR-149 rs2292832 polymorphism and cancer risk. But the results were not conclusive and consistent. Considering the limits of the single study, we performed this meta-analysis of 12 published studies to derive a more powerful estimation of the association between the hsa-miR-149 rs2292832 polymorphism and cancer risk.

## Methods

### Publication search and inclusion criteria

Medical subheading (Mesh) terms: ‘microRNA’, ‘cancer’ and ‘polymorphism’ were used to search on PubMed for eligible studies (last search: April 28, 2013). The references of articles and reviews were also examined to explore potentially additional studies. Studies were eligible if they met the following criteria: (a) case-control studies; (b) investigating the association between the hsa-miR-149 rs2292832 polymorphism and cancer risk; (c) detailed genotype data for estimating of odds ratio (OR) and 95% confidence interval (CI); (d) full text articles in English. If multiple studies had overlapping or duplicate data, only those with complete data or recent studies were included.

### Data extraction

Data were evaluated and extracted from the eligible studies by two investigators (Xu and Zhou) independently. The following items from each study were recorded: first author’s name, year of publication, country or area of origin, ethnicity, cancer type, source of controls, genotyping method, total number of cases and controls, genotype distributions of cases and controls, and Hardy-Winberg equilibrium (HWE), respectively. If discrepancies existed between two investigators, another investigator (Qiu) was invited to discuss and check the data until a consensus was reached.

### Statistical analysis

HWE was evaluated for controls in each study by the chi-square test and a p<0.05 was considered as departure from HWE. Odds ratios (ORs) with 95% confidence intervals (CIs) were used to assess the strength of association between the hsa-miR-149 rs2292832 polymorphism and cancer risk. Pooled ORs were performed for allelic comparison (T vs. C), recessive model (TT vs. CT/CC), dominant model (TT/CT vs. CC), homozygote comparison (TT vs. CC), and heterozygote comparison (CT vs. CC), respectively. The statistic significance of pooled ORs was determined by Z-test and a p<0.05 was considered as statistically significant. A chi-square based Q-test was used to check the heterogeneity among the studies. A p<0.10 for Q-test suggested significant heterogeneity among studies, and the random-effects model (DerSimonian-Laird method) was conducted to calculate the pooled ORs [13]; Otherwise, the fixed-effects model (Mantel-Haenszel method) was used [14]. Subgroup analyses were also performed to test the effects of ethnicity, cancer type and source of controls. Sensitivity analysis was carried out to identify the effect of data from each study on pooled ORs. Begg’s funnel plot and the Egger’s linear regression test were performed to evaluate publication bias of literatures and a p<0.05 was considered significant [15]. Trim and fill method was used to assesses potential asymmetry in the funnel plot. All of the statistical tests were calculated with STATA software version 12.0 (STATA Corporation, College Station, TX, USA).

## Results

### Study characteristics

**Figure 1:**
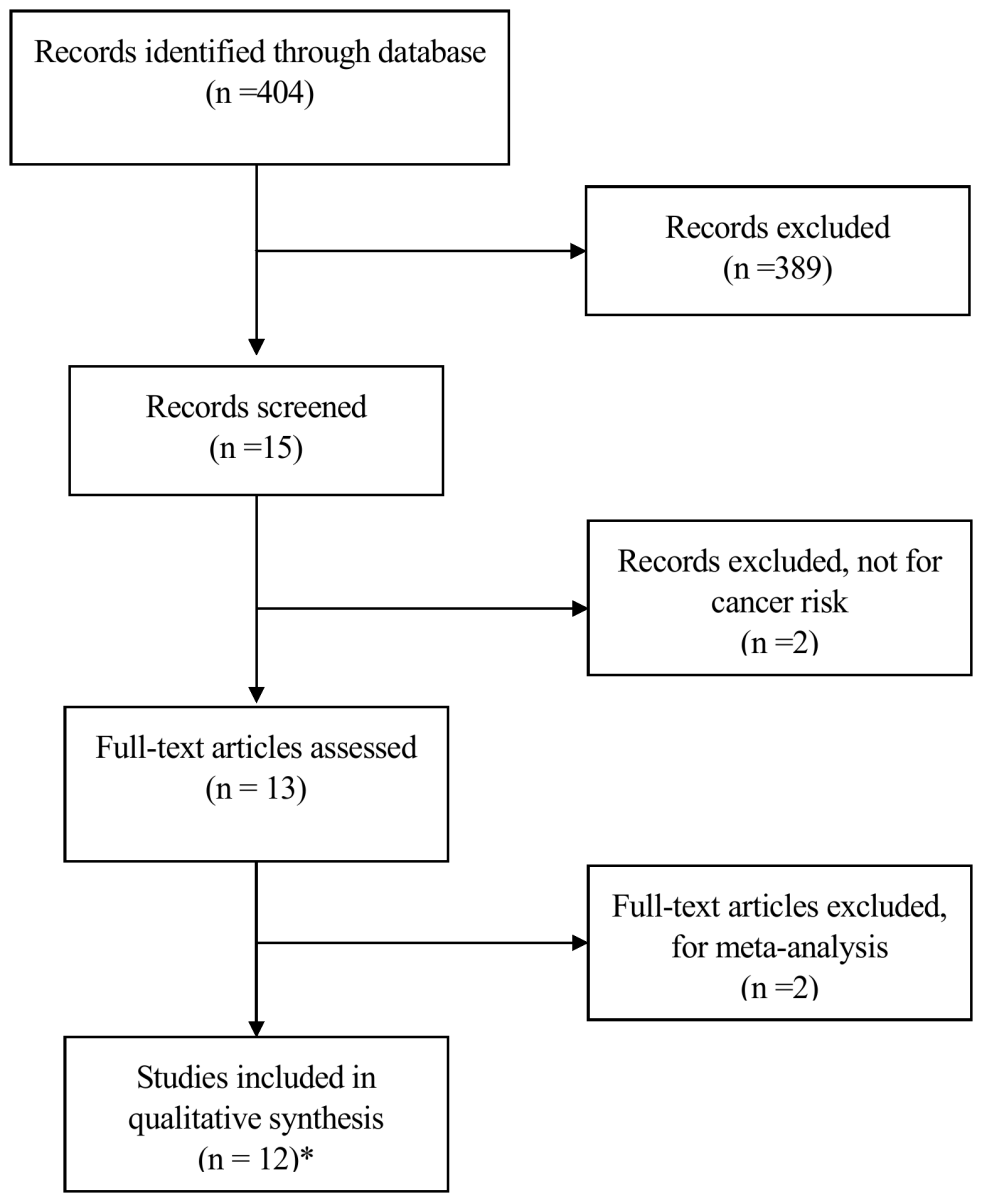
Flow chart of studies in the analysis. *11 articles were retrieved and two separate studies were reported in one article, so 12 studies were eligible.

A total of 11 articles were retrieved from PubMed according to the inclusion criteria [16–26]. Figure 1 shows the detailed screening process. The study of Zhang et al. [21] presented separate OR by different cancer types (gastric cancer and colorectal cancer) and each of them was considered separately in this meta-analysis. Thus, a total of 12 studies involving 5937 cases and 6081 controls were analyzed in our meta-analysis. As shown in Table 1, 3 of 12 studies were Caucasians and the other 9 studies were Asians. Almost all cases were diagnosed histologically or pathologically. High Resolution Melting (HRM), TaqMan genotyping assay and polymerase chain reaction-restriction fragment length polymorphism (PCR–RFLP) were used as genotyping methods in 2, 1 and 9 studies respectively. All studies used blood sample for genotyping. Age and sex were matched for controls in almost all studies, of which 7 were hospital-based and 5 were population-based. The distribution of genotypes in the controls was in agreement with the HWE except the study of Chu et al. [17].

**Table 1:** Characteristics of eligible studies.

**Author**	**Year**	**Country**	**Ethnicity**	**Cancer type**	**Study design**	**Genotyping method**	**P_hwe_**	**Cases**	**Controls**	**Cases**			**Controls**		
										TT	CT	CC	TT	CT	CC
Vinci[16]	2013	Italy	Caucasian	CRC	HB	HRM	0.91	160	178	23	58	79	17	75	86
Chu[17]	2012	Taiwan	Asian	OSCC	HB	TaqMan	<0.001	480	425	345	88	47	315	84	26
Kim[18]	2012	Korea	Asian	HCC	HB	PCR–RFLP	0.34	159	201	81	64	14	83	97	21
Zhang[19]	2012	China	Asian	BC	PB	PCR–RFLP	0.21	245	229	120	102	23	92	113	24
Min[20]	2012	Korea	Asian	CRC	HB	PCR–RFLP	0.95	446	502	221	177	48	232	219	51
Zhang[21]	2012	China	Asian	CRC	PB	PCR–RFLP	0.58	435	443	187	202	46	203	190	50
Zhang[21]	2012	China	Asian	GC	PB	PCR–RFLP	0.70	274	269	132	101	41	114	120	35
Vinci[22]	2011	Italy	Caucasian	LC	HB	HRM	0.97	101	129	16	41	44	11	53	65
Liu[23]	2010	USA	Caucasian	HNSCC	HB	PCR–RFLP	0.27	1109	1130	88	441	580	99	445	586
Tian[24]	2009	China	Asian	LC	PB	PCR–RFLP	0.86	1058	1035	463	472	123	470	453	112
Hu[25]	2009	China	Asian	BC	PB	PCR–RFLP	0.16	1009	1093	99	460	450	108	503	482
Ahn[26]	2012	Korea	Asian	GC	HB	PCR–RFLP	0.98	461	447	241	176	44	220	187	40

CRC: colorectal cancer; OSCC: oral squamous cell carcinoma; HCC: hepatocellular carcinoma; BC: breast cancer; GC: gastric cancer; LC: lung cancer; HNSCC: squamous cell carcinoma of the head and neck PB: population-based; HB: hospital-based. P_hwe_: Hardy-Winberg equilibrium

### Meta-analysis results

The main results of this meta-analysis are listed in Table 2. We did not find any significant association between the hsa-miR-149 rs2292832 polymorphism and cancer risk in all genetic models (T versus C:OR = 1.01, 95% CI 0.95–1.06, P_heterogeneity_ = 0.286; TT vs. CC:OR= 0.98, 95% CI 0.86–1.11, P_heterogeneity_ = 0.451; CT vs. CC:OR= 0.96, 95% CI 0.86–1.05, P_heterogeneity_ = 0.851; TT/CT vs. CC:OR= 0.97, 95% CI 0.88–1.06, P_heterogeneity_ = 0.858; TT vs. CT/CC: OR= 1.05, 95% CI 0.96–1.14, P_heterogeneity_ = 0.118) (see Figure S1 to Figure S5 in File S1). Similarly, no significant association between the hsa-miR-149 rs2292832 polymorphism and cancer risk was found in our subgroup analysis by ethnicity (Asian and Caucasian), study design (hospital-based and population-based) or cancer type (squamous cancer, breast cancer, colorectal cancer, lung cancer and gastric cancer).

**Table 2:** Meta-analysis results.

	**N**	**case/control**	**T vs. C**		**TT vs. CC**		**CT vs. CC**		**TT/CT vs. CC**		**TT vs. CT/CC**	
			**OR**	**Ph**	**OR**	**Ph**	**OR**	**Ph**	**OR**	**Ph**	**OR**	**P_h_**
Total	12	5937/6081	1.01(0.95,1.06)	0.286	0.98(0.86,1.11)	0.451	0.95(0.86,1.05)	0.851	0.97(0.88,1.06)	0.858	1.05(0.96,1.14)	0.118
Cancer type												
SC	2	1589/1555	0.93(0.83,1.05)	0.218	0.8(0.62,1.04)	0.191	0.82(0.49,1.37)	0.07	0.81(0.51,1.3)	0.066	0.89(0.73,1.1)	0.978
BC	2	1254/1322	1.08(0.86,1.35)	0.121	1.04(0.79,1.37)	0.361	0.98(0.82,1.16)	0.907	0.99(0.84,1.17)	0.656	1.17(0.82,1.27)	0.123
CRC	3	1041/1123	1.02(0.9,1.16)	0.669	1.07(0.81,1.43)	0.621	0.94(0.73,1.22)	0.548	0.99(0.77,1.26)	0.889	1.05(0.88,1.25)	0.186
LC	2	1159/1164	1.09(0.76,1.56)	0.073	1.26(0.55,2.9)	0.058	0.99(0.76,1.27)	0.562	0.99(0.78,1.27)	0.238	1.23(0.6,2.54)	0.071
GC	2	735/716	1.07(0.92,1.25)	0.876	0.99(0.7,1.4)	0.983	0.79(0.56,1.12)	0.628	0.89(0.64,1.24)	0.787	1.18(0.96,1.45)	0.608
Study design												
PB	5	3021/3069	0.99(0.92,1.07)	0.402	0.98(0.82,1.16)	0.842	0.97(0.84,1.11)	0.755	0.97(0.85,1.11)	0.924	1.01(0.9,1.13)	0.147
HB	7	2916/3012	1.02(0.94,1.11)	0.182	0.98(0.82,1.18)	0.147	0.94(0.82,1.07)	0.631	0.96(0.85,1.09)	0.505	1.1(0.97,1.24)	0.172
Ethnicity												
Asian	9	4567/4644	1.01(0.94,1.07)	0.26	0.96(0.83,1.1)	0.648	0.93(0.82,1.05)	0.742	0.95(0.84,1.06)	0.786	1.05(0.96,1.14)	0.166
Caucasian	3	1370/1437	1.01(0.9,1.14)	0.22	1.05(0.81,1.38)	0.102	0.99(0.85,1.16)	0.688	1(0.86,1.16)	0.567	1.29(0.76,2.17)	0.082

N: number of studies included; OR: odds ratio; P_h_: p value for heterogeneity; SC: squamous cell carcinoma CRC, colorectal cancer; BC: breast cancer; GC: gastric cancer; LC: lung cancer; PB: population-based; HB: hospital-based

### Sensitivity analysis

One single study was deleted each time from pooled results to investigate the influence of individual study on the pooled ORs. Our results showed that pooled ORs were not altered which suggested that no individual study significantly affected the pooled results.

### Publication bias

**Figure 2:**
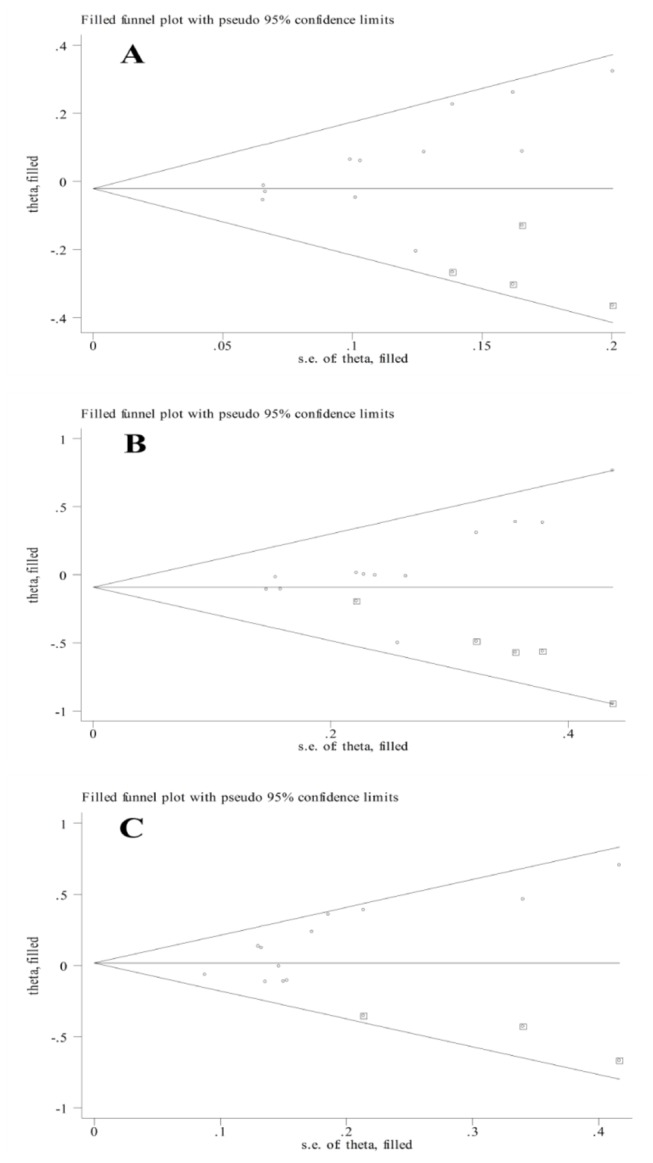
Funnel plot adjusted with trim and fill method (A:T vs. C; B: TT vs. CC; C: TT vs. **CT**/**CC** ). Circles: included studies. Diamonds: presumed missing studies.

We used Begg’s funnel plot and the Egger’s linear regression test to assess publication bias. Publication bias was detected (T vs. C:P = 0.047 for Begg’s test and P=0.025 for Egger’s test; TT vs. CC:P = 0.011 for Begg’s test and P=0.028 for Egger’s test; TT vs. CT/CC: P=0.024 for Begg’s test and P=0.007 for Egger’s test). Thus, a trim and fill method was used and pooled ORs were recalculated with hypothetically non-published to evaluate the asymmetry in the funnel plot (Figure 2). The recalculated ORs did not change significantly (T vs. C:OR = 0.981, 95% CI= 0.914–1.053; TT vs. CC:OR = 0.912, 95% CI= 0.814–1.022; TT vs. CT/CC: OR= 1.031, 95% CI =0.917–1.16), indicating the stability of the results.

## Discussion

MiRNAs play important role in various cellular processes and are involved in many diseases including various cancers [2,27,28]. Genetic variations which arise in miRNA genes including their pri- and pre-miRNA regions, might affect processing and expression of miRNA such as occurs in the down regulation of mature let-7e by a G to A mutation at 19nt downstream of the pre-let-7e [29]. Recently, many studies have demonstrated the association between SNPs of miRNA gene and cancer risk. The miR-27a polymorphism (rs11671784) may decrease the expression of mature miR-27a and reduce susceptibility to gastric cancer [30]. A significantly increased risk of colorectal cancer was observed with the miR-196a2 (rs11614913) CT/CC genotype compared with the TT genotype [31].

As a tumor suppressor gene, miR-149 may inhibit proliferation and induce cell cycle arrest by targeting ZBTB2 in gastric cancer [32]. Pan et al. showed that miR-149 might be involved in the proliferation and invasion of glioma cells via blockade of AKT1 signaling [33]. Thus, alterations in miR-149 gene may contribute to cancer risk. Association between the hsa-miR-149 rs2292832 polymorphism and cancer risk has been explored in some studies. Kim et al. demonstrated that the risk of HCC was significantly lower for patients with the miR-149 CT or CT/CC genotypes [18]. A potentially protective role of C allele of hsa-miR-149 was observed in male gastric cancer [21,26]. But, no significant association between the hsa-miR-149 rs2292832 polymorphism and cancer risk was observed among other studies. As for the inconsistent results from individual studies for all types of cancers, this meta-analysis including 12 studies with 5937 cases and 6081 controls in total was built to assess the association. However, our results showed that no significant association between the hsa-miR-149 rs2292832 polymorphism and susceptibility to cancer. Similarly, subgroup analyses by cancer type, study design or ethnicity did not suggest a significantly different result. When we deleted Chu’s study [17] nonconformity to HWE in the control group in the sensitivity analysis, the pooled results did not change significantly. As publication bias was observed, we adopted trim and fill method to recalculate the adjusted ORs and did not find a different result, suggesting the stability of the statistic analysis.

Several limitations of the meta-analysis should be considered. Firstly, the studies searched on PubMed were full text in English. This may be partially responsible for the observed publication bias, though they do not change the results by using trim and fill method. Secondly, the number of studies for subgroup analysis was small that only one study investigated the association in oral squamous cell carcinoma [17], squamous cell carcinoma of the head and neck [23] and hepatocellular carcinoma [18] respectively, of which Kim’s study [18] revealed biologic significance. More studies with homogeneous cancer patients and controls were needed to confirm the results. Some covariates such as sex, age and residence area were also not available in all studies for adjusted ORs which need to be further considered, as we found the potentially protective role of C allele in male population of gastric cancer.

In conclusion, the pooled results of this meta-analysis suggested that the hsa-miR-149 rs2292832 polymorphism may not contribute to the susceptibility of all types of cancers in spite of the potentially protective role of C allele in hepatocellular carcinoma and male gastric cancer. However, our results should be considered with caution due to the observed publication bias and limitations listed above. To further confirm the results, large scale case-control studies with different ethnic groups and multiple cancer types are needed.

## Supporting Information

File S1 Forest plots of the association between the hsa-miR-149 rs2292832 polymorphism and cancer risk.
(DOCX)

Table S1 **PRISMA checklist.**
(DOC)
