# Supplementary material for: Lack of Association between Hsa-miR-149 rs2292832 Polymorphism and Cancer Risk: A Meta-Analysis of 12 Studies
Source: PLoS One. 2013 Sep 5;8(9):e73762. doi: 10.1371/journal.pone.0073762 (PMC3764043; doi:10.1371/journal.pone.0073762)

Figure S1 Forest plots of the association between hsa-miR-149 rs2292832 polymorphism and cancer risk (T vs. C)


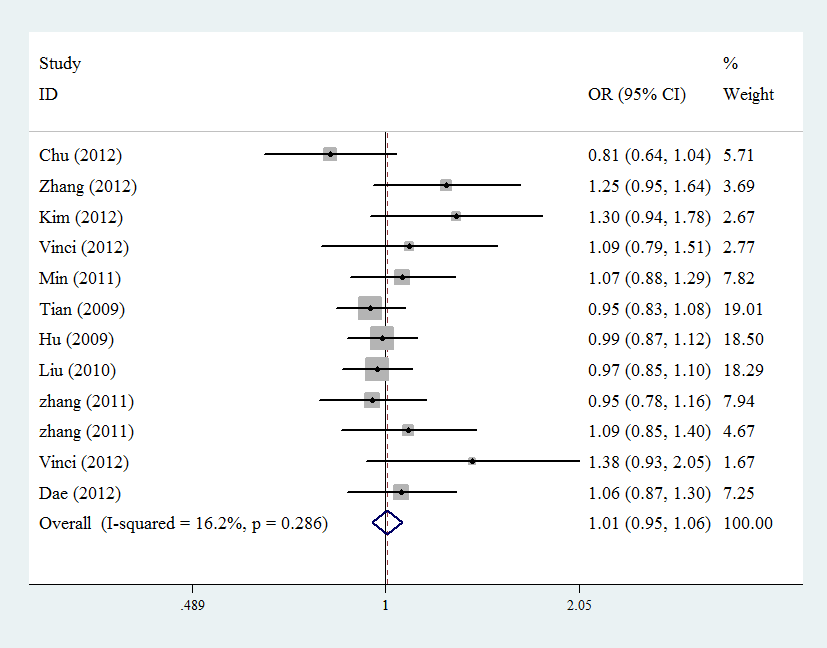


Figure S2 Forest plots of the association between hsa-miR-149 rs2292832 polymorphism and cancer risk under homozygote comparison (TT vs. CC)


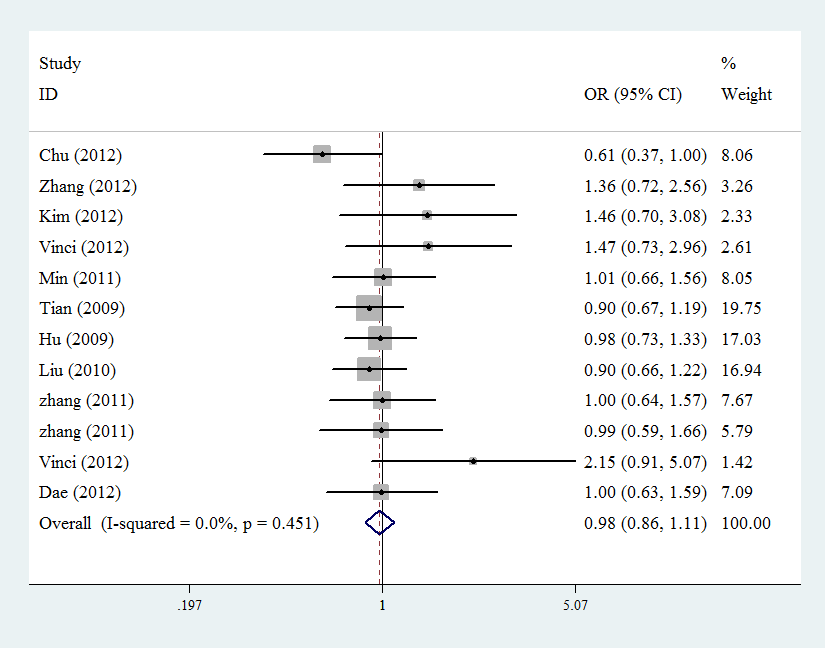


Figure S3 Forest plots of the association between hsa-miR-149 rs2292832 polymorphism and cancer risk under heterozygote comparison (CT vs. CC)


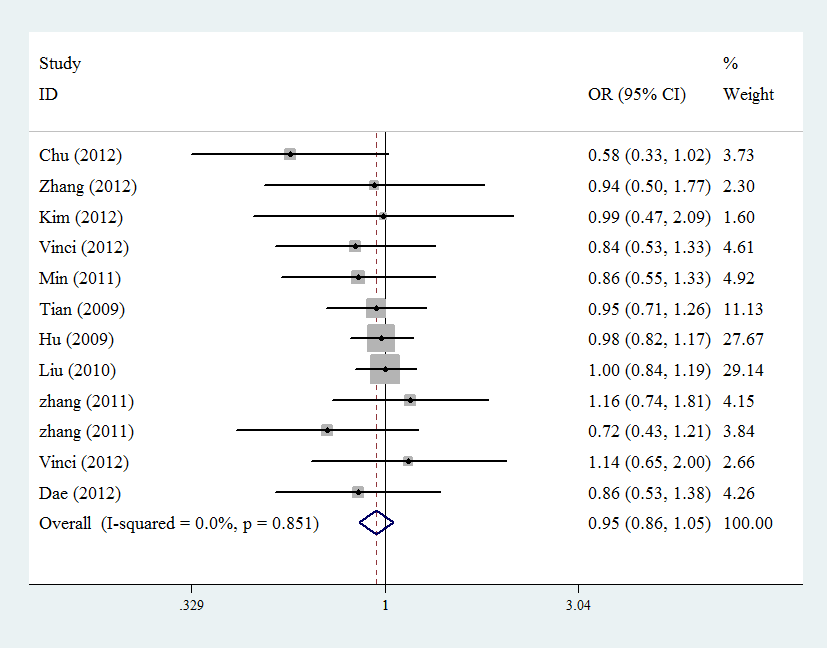


Figure S4 Forest plots of the association between hsa-miR-149 rs2292832 polymorphism and cancer risk in recessive model (TT vs. CT/CC)


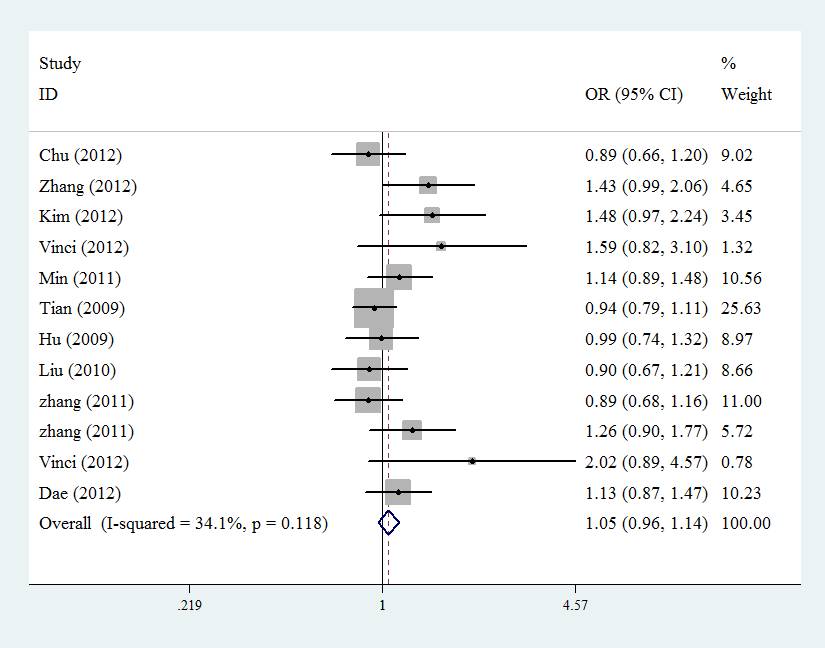


Figure S5 Forest plots of the association between hsa-miR-149 rs2292832 polymorphism and cancer risk in recessive model (TT/CT vs. CC)


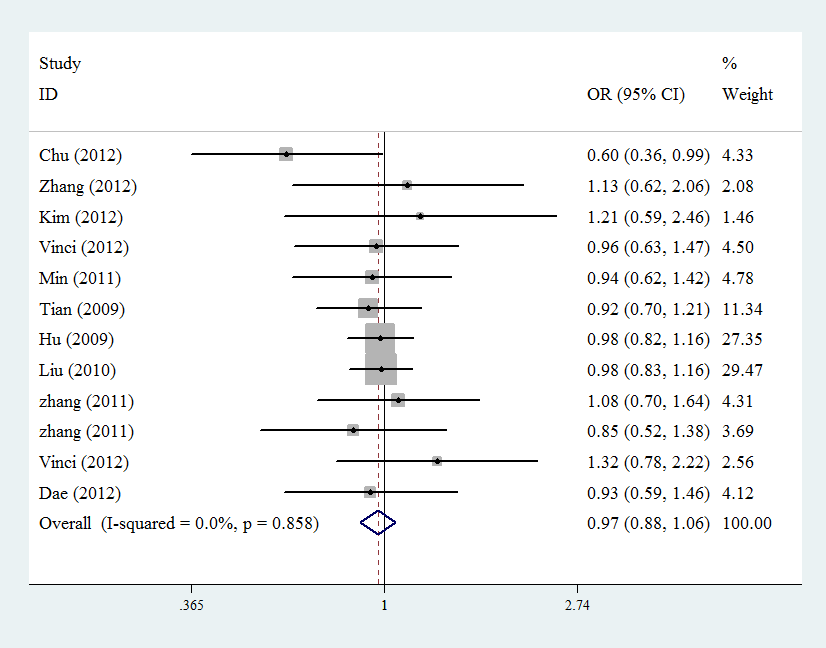

Supplement: File S1 — Forest plots of the association between the hsa-miR-149 rs2292832 polymorphism and cancer risk. (DOCX) [file pone.0073762.s001.docx]
